# Technology-free predictors of preterm birth in singleton women with threatened preterm labor: a prospective cohort study

**DOI:** 10.1186/s12884-022-05155-3

**Published:** 2022-11-08

**Authors:** Maryam Najjarzadeha, Sakineh Mohammad-Alizadeh-Charandabi, Shamsi Abbasalizadeh, Mohammad Asghari-Jafarabadi, Mojgan Mirghafourvand

**Affiliations:** 1 Department of Midwifery, Faculty of Nursing and Midwifery, Umia University of Medical Sciences, Urmia, Iran; 2 Social Determinants of Health Research Center, Department of Midwifery, Faculty of Nursing and Midwifery, Tabriz University of Medical Sciences, Shariatie Ave, Tabriz, Iran; 3 Women’s Reproductive Health Research Center, Tabriz University of Medical Sciences, Tabriz, Iran; 4 Cabrini Research, Cabrini Health, Melbourne, VIC 3144 Australia; 5 School of Public Health and Preventative Medicine, Faculty of Medicine, Nursing and Health Sciences, Monash University, Melbourne, VIC 3800 Australia; 6 Road Traffic Injury Research Center, Tabriz University of Medical Sciences, Tabriz, Iran; 7 Menopause Andropause Research Center, Ahvaz Jundishapur University of Medical Sciences, Ahvaz, Iran

**Keywords:** Premature birth, Preterm birth, Predictor, Technology-free, Threatened preterm labor

## Abstract

**Background:**

Prediction of preterm birth is still a challenge due to its multiple etiologies. This prospective cohort study aimed to determine the technology-free predictors of preterm birth in singleton women with threatened preterm labor.

**Methods:**

This prospective cohort study was performed on 371 singleton women with gestational age of 23^+ 6^ to 36^+ 4^ weeks hospitalized for threatened preterm labor. The data were collected using a questionnaire including demographic characteristics, medical and maternal history, as well as the Perceived Stress Scale (PSS), the Multidimensional Scale of Perceived Social Support (MSPSS), and the WHO’s questionnaire of Violence against Women (VAW). The participants were followed-up until childbirth. The predictors were determined using multivariate logistic regression.

**Results:**

Preterm birth occurred in 51% of women. Seven variables were determined as predictors; rupture of membranes [adjusted odds ratio 11.7, 95% confidence interval 5.4 to 25.6], cervical dilation [AOR 4.1, 95% CI 2.0 to 7.0], gravidity ≥6 [AOR 27.4, 95%CI 2.8 to 264.3], psychological violence during pregnancy [AOR 2.0, 95% CI 1.1 to 3.2], medical problems in pregnancy onset [AOR 1.7, 95% CI 1.1 to 2.8], vaginal bleeding/spotting [AOR 2.1, 95% CI 1.2 to 4.0] and woman age ≤ 19 [AOR 2.2, 95% CI 1.1 to 4.5]. The proportion of variance explained by all these factors was 33.6%.

**Conclusions:**

The technology-free factors seems to have moderate power in preterm birth prediction in singleton pregnant women hospitalized for threatened preterm labor. However, these results are discoveries without verification or validation and need to be confirmed by generalizable studies.

## Background

Premature birth (birth < 37 completed weeks of gestation) is an adverse health outcome and can be associated with severe life-long disabilities of the infant [1]. According to the Global Burden of Disease, this outcome has the 8th rank of the disability adjusted life years (DALYs) [2]. Reported prevalence of preterm birth is 11% of live births in the world [3] and 10% of live births in Iran [4].

Clinical professionals should estimate and predict the likelihood of adverse health outcomes [5] to be able to reduce the preventable part of the outcomes and their subsequent problems. There are multiple etiologies leading to preterm birth. All these factors lead to decidua and membrane activation and finally uterine contraction and cervical ripening. Main part of these factors such as stress, inflammation or infection, uterine distension are preventable [6]. Also, some of the socioeconomic, environment and behavioral and life style factors can be controlled [6, 7]. However, preterm birth prediction [8, 9] and prevention is still a global challenge [6, 10, 11].

Studies on predictors of premature birth in women with threatened preterm labor often rely on biomarkers such as placental alpha-microglobulin-1 and fetal fibronectin using the sophisticated laboratory methods such as immunoassay [12, 13]. Beside their high cost, these methods cannot be performed in all laboratories [12]. Therefore, such approaches cannot be extensively performed in most of the world, particularly in developing countries [14]. Therefore, development of predicting models using measurable inexpensive factors without the need for advanced technologies should be addressed in most regions of the world, especially in low- and middle-income countries [15].

Some studies have been performed to determine technology-free measurable predictive factors or to develop risk scoring scale tool for predicting preterm birth [7, 15–17]. However, we found only one study on the preterm birth predictors conducted on women with threatened preterm labor in a high income country [18]. Therefore, this study aimed to determine demographic, medical, maternal, and psychological predictors of preterm birth in women with threatened preterm labor in Iran (a middle-income country).

## Methods

### Study design and setting

This study is part of a hospital-based cohort study entitled “Risk factors and predictors of preterm birth in women with threatened preterm labor.” In this prospective cohort study, the samples were enrolled from two tertiary maternity hospitals (Alzahra and Taleghani) in Tabriz, Iran, from June 22, 2019 to July 28, 2020 and follow-up ended at October 15, 2020. Tabriz (the capital of East Azerbaijan province) is a referral city in the northwest of Iran in terms of facilities for specialized care of preterm neonates, and almost all women threatened with preterm labor at less than 32 weeks of gestation are referred to the Alzhara hospital from other cities of the province and sometimes from the neighbor provinces.

### Sampling and participants

Convenience consecutive sampling was performed among all the eligible women in the research setting. The study population included all singleton women at the gestational age of 23^+ 6^ to 36^+ 4^ weeks with live and healthy fetus who were hospitalized at the hospitals with signs and symptoms of threatened preterm labor. Signs and symptoms of threatened preterm labor were defined as regular and painful uterine contractions (at least one elevation in baseline tone with a rounded peak lasting 40-120 seconds in Tocogram monitoring during 10 minutes) or changes in cervical dilatation and effacement or preterm rupture of membranes. Uterine contractions were detected by Tocogram in cardiotocography. We did not include women with cervical insufficiency or short cervix in the study who did not met the above criteria. Illiterate women, and those who themselves or their spouses were not Iranian were excluded.

### Data collection

The questionnaires were coded and identifiable characteristics of the participants along their codes were recorded in a separate sheet. All participants were ensured of confidentiality before the interview and sensitive questions such as sexual activity and sexual violence were placed at the end of questionnaires. Each interview took 35-45 minutes according to the participants’ clinical conditions – frequency and duration of uterine contractions and severity of labor pain. The women’s phone number and national code were obtained to follow-up the birth date through the National Birth Registry System (SIB), if necessary.

The gestational age was determined in the first interview according to results of the ultrasound performed in the 8th to 18th week of pregnancy. Based on the gestational age and the exact delivery date, occurrence of premature birth were objectively determined (prevention of diagnosis bias). To avoid information bias, interviews were performed in a private environment when the participant had no uterine contraction and felt comfortable.

Data pertaining to possible risk factors were collected through face-to-face interviews with all eligible women in the high-risk pregnancy or childbirth wards, within the first 24 hours of hospital admission (after stabilization of their conditions in the admitted ward). The interviews were performed by a highly experienced midwife (first author) in the morning or evening shifts in a private and calm environment. The required follow-up data (including the exact childbirth date in women who discharged before delivery) were obtained through phone interview with the participants.

### Tools

The data were collected using a researcher-made preterm birth risk factors questionnaire included socio-demographic characteristics, medical and maternal history. Other tools were standard questionnaires included, the Cohen’s Perceived Stress Scale (PSS-10), the Zimet’s Multidimensional Scale of Perceived Social Scale (MSPSS-12) and the Violence against Women Questionnaire (WHO-VAW-13). The researcher-made questionnaire developed through literature review based on the integrative method. The content validity of this questionnaire was determined using expert opinions of 10 experts in the Tabriz University of Medical Sciences.

#### PSS-10

The PSS is a standard and international self-report tool used assessment of perceived stress in last month, this scale developed by Cohen et al. [19]. It has 10 items by Likert scoring never to very often (0-4). The total score of PSS was obtained from the sum of all 10 items, the greater scores, the more perceived stress in last month. The validity and reliability of PSS were confirmed, by Khalili et al. in patients with chronic headache, in Iran [20]. The reliability of this scale also was assessed, by using Cronbach’s α and intra-class correlation coefficient (ICC), in pregnant women in this study and the results published before [21]. We defined sum score 20 and more defined as high stress, in our study.

#### MSPSS-12

The Perceived Social Support (PSS-12), is a multidimensional scale which measures perceived social support in three domains; family (4 items), friends (4 items) and other important people (4 items), it first developed by Zimet et al. [22]. The total score of MSPSS was obtained from the sum of all 12 items, the greater scores, the more perceived social support. The validity and reliability of this tool confirmed by Bagherian et al. in myocardial infarction (MI) patients, in Iran [23]. In our study with another group of people, pregnant women threatened to preterm birth, the reliability of MSPSS also was assessed, by using Cronbach’s α and intra-class correlation coefficient (ICC), and the results published before [21]. In this study sum score 40 and more defined as high social support.

#### VAW-13

The WHO-VAW-13 has 13 items with four options (never, once, sometimes, and often) and measures violence in three dimensions: psychological (4 items), physical (6 items) and sexual (3 items). This scale was used in WHO multicenter studies in 10 countries in 2005 [24]. Details of the psychometric results of this scale published in another report [21]. Content validity ratio (CVR), content validity index (CVI), item impact score and reliability of WHO-VAW-13 were assessed in this study. Results showed; with CVR 0.8-1.0 and CVI 0.9-1.0 for all 13 items, WHO-VAW-13, is a valid tool for measuring violence in pregnant women. Also, according by 15 pregnant women comments who participated in lay panel, with item impact scores 4 and more than 4, for all 13 items, the WHO-VAW-13, have sufficient face validity.

In our study answer “yes” for at least one of the 13 items (psychological or physical or sexual violence), defined as experience of any kind of violence, answer “yes” for at least one of the four first items (1-4) defined as experience of psychological violence, answer “yes” for at least one of the six items (5-10) defined as experience of physical violence, answer “yes” for at least one of the next four items (11-13) defined as experience of sexual violence.

### Sample size

To determine predictors using the regression model with six or more predictors, at last 10 and in the ideal conditions 30 participants are required for each predictor [25]. In this study, 371 participants were able to determine at last 13 predictors in the ideal conditions.

### Data analysis

The data were analyzed using SPSS-21 (SPSS, Chicago, IL, USA) and *P* <  0.05 was considered statistically significant. Quality of the inserted data was controlled by randomly rechecking 10% of the data and frequency checking. The predictors were determined using the multivariable binary logistic regression with the backward stepwise strategy, by inserting factors that were related to preterm birth in the unadjusted binary logistic regression with *P* <  0.2. The goodness of fit of the model was investigated using the Hosmer and Lemeshow test. The Nagelkerkes R square was used to measure the proportion of total variance predicted by the models. Variance inflation factor (VIF) more than five was considered as a sign of high multicollinearity between independent variables. In this study, *p* <  0.05 was considered to be significant.

## Results

Out of 465 women approached, 371 were enrolled in the study and 94 were excluded due to multiple pregnancy (*n* = 38), uterine anomalies (*n* = 9), vaginal bleeding due to placental problems (*n* = 8), suspicion of chorioamnionitis (*n* = 7), uterine cerclage or cervical insufficiency or shortness (*n* = 7), unwillingness to participate in the study (*n* = 13), and illiteracy (*n* = 12). There was no loss to follow-up, and the data of all 371 enrolled eligible women were analyzed.

The mean age of participants was 27.4 (SD 6.8) years. About 75% of them had a high school diploma or lower. Most of the women (73%) were living in the urban areas and 82% were housewives. The majority of the participants were primi-gravida (40%) or second gravida (31%). Mean gestational age at admission was 31^+ 5^ (SD 2^+ 6)^ weeks. One hundred eighty-eight women (51%) gave birth before completion of the 37th week of gestation. Also some socio-demographic and obstetrics characteristic of the participants who delivered before and after 37 weeks mentioned in Table 1.

**Table 1:** Socio-demographic and obstetrics characteristic of the participants in the study (*N* = 371)

Characteristics^**a**^	Delivery < 37 weeks (***n*** = 188)		Delivery ≥ 37 weeks (***n*** = 183)
**Age (years)**			
≤ 19	36 (19.1)		19 (10.4)
20-34	116 (61.7)		130 (71.0)
≥ 35	36 (19.1)		34 (18.6)
**Level of education**			
Primary	34 (18.1)		31(16.9)
Secondary and diploma	107 (56.9)		107 (58.5)
Academic	47 (25.0)		45 (24.6)
**Job**			
Housekeeper	155 (82.4)		148 (80.9)
Employed	33 (17.6)		35 (19.1)
**Household monthly income**			
Sufficient	52 (27.6)		53 (29.0)
Partly sufficient	97 (51.7)		94 (51.3)
Insufficient	39 (20.7)		36 (19.7)
**Living Place**			
Rural area	53 (28.2)		46 (25.1)
Urban area	135 (71.8)		137 (74.9)
**Gravida**			
1	74 (39.4)		76 (41.5)
2	58 (30.8)		59 (32.3)
3 or more	56 (29.8)		48 (26.2)
**GA at admission (wk)**^**b**^			
< 28	27 (14.4)		14 (7.7)
28-33^4^	57 (30.3)		61 (33.3)
33^5^-36^4^	104 (55.3)		108 (59.0)

^a^ Results are number (percent)

^b^ Gestational age in weeks and day

In unadjusted analyses, four factors were significantly associated with increased risk of preterm birth including maternal age of ≤19 years, history of spotting or bleeding during the current pregnancy, rupture of membranes and cervical dilation (*p* <  0.05). These factors along with 12 additional factors which were associated with the risk of preterm birth with *p* < 0.2 were entered in the multivariate logistic analysis. The additional factors included; gravidity of ≥6, history of premature birth in mother, gestational age less than 28 weeks at admission, experience of psychological or physical violence during pregnancy, abnormal result(s) in routine early pregnancy tests (those who had at least one abnormal result in routine early pregnancy tests such as thyroid stimulating hormone, fasting blood sugar, complete blood count, urine analysis and others), less than normal weight gain during pregnancy (normal weight gain (kg) during pregnancy based on pre-pregnancy BMI; less than 18.5 kg/m2: 12.5 – 18, between 18.5 and 24.9 kg/m2: 11.5 – 16, between 25 and 29.9 kg/m2: 7 – 11.5, greater than 30 kg/m2: 5 – 9), sexual intercourse during last week before admission, leukocytosis of ≥16,000 at admission, vaginal bleeding or spotting at admission, uterine contraction, high perceived stress (Tables 2, 3, 4, totally 16 factors). Experience of any kind of violence during pregnancy (“yes” for at least one of the 13 items defined as experience of any kind of violence) didn’t enter in the multivariate model, because of high multicollinearity (VIF = 6.6) with other kind of violence.

**Table 2:** Association of some demographic variables with preterm delivery before 37 weeks in singleton pregnancies (*N* = 371)

Variable	N	Preterm birth (***n*** = 188)		
		n (%)	cOR (95% CI)^**a**^	P^**b**^
**Woman age (years)**				
≤ 19	55	36 (65.5)	2.12 (1.15 to 3.90)	0.015
20-34	246	116 (47.2)	Reference	
≥ 35	70	36 (51.4)	1.20 (0.70 to 2.02)	0.528
**Woman education**				
Primary	65	34 (52.3)	1.04 (0.64 to 1.70)	0.862
Secondary/diploma	214	107 (50.0)	Reference	
Academic	92	47 (51.1)	1.10 (0.63 to 1.91)	0.745
**Woman employment**				
Employed	68	33 (48.5)	Reference	
Unemployed	303	155 (51.2)	1.11 (0.65 to 1.88)	0.696
**Household income**				
Sufficient/Partly Sufficient	296	149 (50.3)	Reference	
Insufficient	75	39 (52.0)	1.07 (0.64 to 1.77)	0.797
**Living location**				
Urban	272	135 (49.6)	Reference	
Rural	99	53 (53.5)	1.17 (0.73 to 1.85)	0.506

^a^ Crude Odds Ratio

^b^ Using unadjusted logistic regression

**Table 3:** Association of some obstetrics variables with preterm delivery before 37 weeks in singleton pregnancies (*N* = 371)

Variable	N	Preterm birth (***n*** = 188)		
		n (%)	cOR (95% CI)^**a**^	P^**b**^
**Gravida**				
Primigravida	150	74 (49.3)	0.96 (0.63 to 1.46)	0.866
2–5	213	107 (50.2)	Reference	
6 or more	8	7 (87.5)	6.93 (0.84 to 57.34)	0.072
**Previous Caesarean section**				
No	295	152 (51.5)	Reference	
Yes	76	36 (47.4)	0.85 (0.51 to 1.40)	0.518
**Last childbirth interval**^**c**^				
> 12 months	160	79 (49.4)	Reference	
≤ 12 months	22	14 (63.6)	1.80 (0.71 to 4.51)	0.214
**Previous abortion**				
No	266	131 (49.2)	Reference	
Yes	105	57 (54.3)	1.22 (0.79 to 1.92)	0.382
**History of preterm birth**				
No	312	152 (48.7)	Reference	
Yes	59	36 (61.0)	1.65 (0.93 to 2.91)	0.085
**Gestational age (weeks)**				
> 32	212	104 (49.1)	Reference	
28 - 32	118	57 (48.3)	0.97 (0.62 to 1.52)	0.896
< 28	41	27 (65.9)	2.00 (0.99 to 4.03)	0.052
**Spontaneous pregnancy**				
Yes	309	152 (49.2)	Reference	
No	62	36 (58.1)	1.43 (0.82 to 2.48)	0.204
**Abnormal result(s) in routine early pregnancy tests**				
No	214	102 (47.7)	Reference	
Yes	157	86 (54.8)	1.33 (0.88 to 2.01)	0.176
**Weight gain during pregnancy**				
Normal	76	34 (44.7)	Reference	
Less than normal	239	128 (53.6)	1.42 (0.85 to 2.39)	0.181
Over normal	56	26 (46.4)	1.07 (0.53 to 2.14)	0.847
**Physical trauma during pregnancy**				
No	330	166 (50.3)	Reference	
Yes	41	22 (53.7)	1.14 (0.60 to 2.19)	0.685
**Infected disease during pregnancy**				
No	58	29 (50.0)	Reference	
Yes	313	159 (50.8)	1.03 (0.59 to 1.81)	0.911
**Thyroid disorder during pregnancy**				
No	330	165 (50.0)	Reference	
Yes	41	23 (56.1)	1.28 (0.66 to 2.45)	0.462
**Sexual intercourse during last week before admission**				
No	258	138 (53.5)	Reference	
Yes	113	50 (44.2)	0.69 (0.44 to 1.07)	0.102
**History of vaginal bleeding or spotting during the current pregnancy**				
No	221	102 (46.2)	Reference	
Yes	150	86 (57.3)	1.57 (1.03 to 2.38)	0.035
**Leukocytosis at admission**				
No	338	167 (49.4)	Reference	
Yes	33	21 (63.6)	1.79 (0.85 to 3.76)	0.123
**Hemoglobin**				
≥ 11	327	165 (50.5)	Reference	
< 11	44	23 (52.3)	1.07 (0.57 to 2.02)	0.821
**Vaginal bleeding or spotting at admission**				
No	291	142 (48.8)	Reference	
Yes	80	46 (57.5)	1.42 (0.86 to 2.34)	0.169
**Rupture of membranes at admission**				
No	296	123 (41.6)	Reference	
Yes	75	65 (86.7)	9.14 (4.52 to 18.50)	< 0.001
**Cervical dilatation**				
No	135	45 (38.9)	Reference	
Yes	236	143 (60.6)	3.07 (1.97 to 4.79)	< 0.001
**Uterine contraction**				
No	95	41 (43.2)	Reference	
Yes	276	147 (53.3)	1.50 (0.94 to 2.40)	0.090

^a^ Crude Odds Ratio

^b^ Using unadjusted logistic regression

^c^ One hundred eighty-nines were nulliparous

**Table 4:** Association of some psychological variables with preterm delivery before 37 weeks in singleton pregnancies (*N* = 371)

Variable	N	Preterm birth (***n*** = 188)		
		n (%)	cOR (95% CI)^**a**^	P^**b**^
**Sleep disorder** ^**c**^				
No	144	72 (50.0)	Reference	
Yes	227	116 (51.1)	1.04 (0.69 to 1.59)	0.836
**High perceived stress**				
No	212	101 (47.6)	Reference	
Yes	159	87 (54.7)	1.33 (0.88 to 2.00)	0.178
**High perceived social support**				
Yes	308	153 (47.9)	Reference	
No	63	35 (55.6)	1.26 (0.73 to 2.18)	0.396
**Experience of violence during current pregnancy** **Any kind of violence**				
No	123	56 (45.5)	Reference	
Yes	248	132 (53.2)	1.36 (0.88 to 2.10)	0.163
**Psychological violence**				
No	138	61 (44.2)	Reference	
Yes	233	127 (54.5)	1.51 (0.99 to 2.31)	0.056
**Physical violence**				
No	296	144 (48.6)	Reference	
Yes	75	44 (58.7)	1.41 (0.86 to 2.32)	0.122
**Sexual violence**				
No	322	164 (50.9)	Reference	
Yes	49	24 (49.0)	0.92 (0.51 to 1.69)	0.799

^a^ Crude Odds Ratio

^b^ Using unadjusted logistic regression

^c^ Mean sleep time less than 7 or more than 9 hours during a day and/ or dissatisfaction with sleep quality during last month

Rupture of membranes [adjusted odds ratio 11.7, 95% confidence interval 5.4 to 25.6], cervical dilation [AOR 4.1, 95% CI 2.0 to 7.0], gravidity ≥6 [AOR 27.4, 95%CI 2.8 to 264.3], psychological violence during pregnancy [AOR 2.0, 95% CI 1.1 to 3.2], medical problems in pregnancy onset [AOR 1.7, 95% CI 1.1 to 2.8], vaginal bleeding/spotting [AOR 2.1, 95% CI 1.2 to 4.0] and woman age ≤ 19 [AOR 2.2, 95% CI 1.1 to 4.5]. The proportion of the variance explained by all these factors was 33.6% (Table 5).

**Table 5:** Predictors of preterm delivery among hospitalized singleton women with threatened preterm labor (188 preterm births out of 371 participants)

Predictors	n (%)	aOR (95% CI)^**a**^	P
**Rupture of membranes at admission**	65 (17.5)	11.7 (5.4 to 25.6)	< 0.001
**Cervical dilatation**	143 (38.5)	4.1 (2.4 to 7.0)	< 0.001
**Grand multigravida (gravidity ≥ 6)**	7 (1.9)	27.4 (2.8 to 264.3)	0.004
**Experience of psychological violence during pregnancy**	127 (34.2)	2.0 (1.1 to 3.2)	0.009
**Vaginal bleeding or spotting at admission**	46 (12.4)	2.1 (1.2 to 4.0)	0.013
**Abnormal result(s) in routine early pregnancy tests**	86 (23.2)	1.7 (1.1 to 2.8)	0.024
**Woman age ≤ 19 years**	36 (9.7)	2.2 (1.1 to 4.5)	0.026

^a^ Using logistic regression adjusted (Adjusted Odds Ratios) for all 16 factors with *p* < 0.2 in the unadjusted analysis. We did not enter experience of any kind of violence in the model because of its high collinearity (VIF = 6.6), with the other kind of violence. Excluded factors; weight gain during pregnancy, gestational age less than 28 weeks at admission, vaginal bleeding during pregnancy, high perceived stress, sexual intercourse during last week before admission, history of preterm birth in mother, leukocytosis, uterine contraction, experience of physical violence during pregnancy. *P* = 0.987 for Hosmer & Lemeshow test of the goodness of fit, Nagelkerkes R^2^ = 0.336

## Discussion

The incidence of premature birth in women with threatened preterm labor was 51%. The most important predictors of preterm birth in the women were rupture of membranes, cervical dilation, and gravidity of ≥6.

The results of this study regarding rupture of membranes and vaginal bleeding or spotting as predictors of preterm birth in women with threatened preterm labor were consistent with results of the study conducted in France by Allouche et al. Based on the study results, they developed and validated a nomogram for prediction of premature birth in women with threatened preterm labor [26].

According to the adjusted analysis, rupture of membranes at admission is a powerful predictor of preterm birth (aOR 11.7). Infection of mother or the fetus, as a precursor of preterm birth, is considered as the cause of 30% of the preterm births. Ascending infection from the vagina is a way of mother or fetus infection [27]. Intact membranes of the fetus serve as a physiological barrier against ascending of infection from the lower parts of the birth canal [28]. After rupture of membranes and destruction of this physiologic barrier, the second precursor of preterm delivery occurs that is the cause of the other 25% of preterm birth [27]. Therefore, it is justifiable to increase the odds of premature birth by about 12 times after rupture of membranes.

Our results showed that vaginal bleeding or spotting at admission increases odds of preterm birth two times. Intrauterine bleeding may result in the initiation of labor through activation of decidua, an acceptable theory for onset of term delivery [27]. Therefore, doubling of the odds of preterm birth in women with vaginal bleeding at admission seems logic. Our results are consistent with Raba et al. [18], and Elias et al. [29] study’s results, which indicated vaginal bleeding during pregnancy in the both first or second trimester is a risk factor for preterm birth. In Elias et al.’s study [29], with Bayesian networks and mediation analysis approach, vaginal bleeding in the first trimester mediated by vaginal bleeding in the second trimester, reported as one of the three identified pathways for occurrence of preterm birth.

In this study, gravidity of ≥6 was an important predictor of preterm delivery. We found no study about the effect of grand multigravida on preterm birth. In a previous study [30] which showed the lack of association of the number of gravidity with preterm birth, the researchers considered the variable as a dichotomous variable (with or without history of previous pregnancies). The results of this study are consistent with those of ours in terms of the lack of a significant difference between primigravida women and those with 2-5 pregnancies. Vink et al. also acknowledged this knowledge gap [31]. Given the low number of women with ≥6 pregnancies in this study (only eight), it seems that to fill this knowledge gap, exact effect of grand multigravida should be assessed in future studies with a higher number of this group.

The reported prevalence of psychological, physical, and sexual violence in this study (i.e. 63, 20, and 13%, respectively) were similar to those reported in previous studies in the northwest of Iran [32, 33]. Although in unadjusted analysis experience of both psychological and physical violence during the present pregnancy were associated with increased risk of preterm birth, only psychological violence was recognized as the predictor of preterm birth. Psychological violence can lead to poor fertility health. In male-dominant societies like Iran, with extensive gender inequality, physical and sexual violence may be less reported due to its social and cultural aversion [33].

The results of this study regarding the adolescent pregnancy, as a predictor of increased risk of preterm birth, are consistent with those of a WHO study performed on a large number of women in 29 countries throughout the world [34]. Reproductive immaturity refers to a condition in which a woman becomes pregnant at a gynecologic age (age from menarche) of less than 3 years. This condition occurs in adolescent pregnant women and predisposes them to premature birth due to not completion of maturity stages, incomplete growth and development of reproductive organs, and short cervix [35].

According to national guidelines in Iran [36], all pregnant women should routinely undergo lab tests usually at 6-10 weeks of gestation (before the onset of physiological changes and pregnancy complications). It is done to determine the presence of some underlying diseases such as thyroid disorders, kidney disease, diabetes, urogenital infections, and anemia in early pregnancy and to initiate appropriate treatment if there is an abnormality. In this study, abnormal result(s) in routine early pregnancy tests which may show the presence of one or more of the mentioned diseases increased the odds of preterm birth by 70% in women with threatened preterm labor. The results of previous studies also showed an increased risk of preterm birth in women with hypothyroidism [37], overt diabetes [38], urogenital infections [18], and anemia in the first trimester of pregnancy [39].

The prospective longitudinal design of this study, relatively high sample size, and no loss to follow-up of participants can be considered as the strengths of the present study. Also, its conduction in a high diverse population could increase the generalizability of the results. The relative low predictive power of the model obtained in this study can be considered as one of the limitations of this study. However, due to the use of measurable data by first-level health professionals to predict the likelihood of preterm delivery, the results can be widely used, especially in less developed countries with no or less access to advanced diagnostic facilities. Given the observational nature of studies, the relationships identified in this study should not be considered as a cause-and-effect relationship. It is also recommended to repeat similar studies in other communities with high cases of grand multigravida to help fill the existing knowledge gap.

## Conclusions

Our study results indicated that rupture of membranes, cervical dilation, gravidity of ≥6, psychological violence during current pregnancy, abnormal result(s) in routine early pregnancy tests, vaginal bleeding or spotting at admission and age of ≤19 are predictors of birth less than 37 weeks of gestation in women with threatened preterm labor. The technology-free factors have a moderate power in prediction of premature birth in the women. However, these results are discoveries without verification or validation and need to be confirmed by generalizable studies.

## Data Availability

The datasets used and analyzed in this study can be made available by the corresponding author at reasonable request.

## Abbreviations

DALYs: Disability adjusted life years
SIB: National Birth Registry System
PSS: Perceived Stress Scale
MSPSS: Multidimensional Scale of Perceived Social Scale
WHO-VAW-13: WHO’ Violence Against Women 13
